# Effects of low-dose lithium supplementation on thermal inactivation of SERCA in cardiac muscle of male mice

**DOI:** 10.1042/BSR20253525

**Published:** 2026-05-13

**Authors:** Sophie I. Hamstra, Mia S. Geromella, Panagiota Klentrou, Peter M. Tiidus, Rebecca E.K. MacPherson, Val A. Fajardo

**Affiliations:** 1 Centre for Bone and Muscle Health, Brock University, St. Catharines, ON L2S 3A1, Canada; 2 Department of Kinesiology, Faculty of Applied Health Sciences, Brock University, St. Catharines, ON L2S 3A1, Canada; 3 Department of Health Sciences, Faculty of Applied Health Sciences, Brock University, St. Catharines, ON L2S 3A1, Canada

**Keywords:** GSK3, Lithium, PLN, SERCA, Thermal inactivation

## Abstract

The sarco(endo)plasmic reticulum calcium (Ca^2+^)-ATPase 2 (SERCA2) is a crucial regulator of cardiac muscle function that is sensitive to changes in the cellular environment, such as increased oxidative stress. This can be observed in heat stress experiments, where the thermal inactivation of SERCA is linked to an increased production of reactive oxygen species. Previous studies have shown that regulatory proteins, including phospholamban (PLN) and heat shock protein 70 (Hsp70), can physically bind to SERCA2, preserving its function in the face of heat stress. Furthermore, we have demonstrated that the inhibition of glycogen synthase kinase 3β (GSK3β) can alter the protein levels of PLN and HSP70 in cardiac tissues obtained from male mice; however, its potential downstream effects on the thermal inactivation of SERCA have not yet been investigated. In the present study, we examined the potential effects of GSK3β inhibition through six weeks of low-dose lithium chloride supplementation (LiCl, 10 mg/kg body mass/day via drinking water) on the thermal inactivation of left ventricle SERCA2 obtained from male C57BL/6J mice. Our results show that LiCl supplementation increased inhibitory serine 9 phosphorylation on GSK3β while also significantly raising SERCA2 content and the SERCA2:PLN ratio. There were no changes to Hsp70 with LiCl supplementation. Although LiCl decreased baseline maximal SERCA activity, the decline in activity in response to heat stress was less compared with control. In conclusion, GSK3β inhibition with LiCl is associated with protection of SERCA from thermal inactivation in the heart, and future studies should explore the underlying cellular mechanisms.

## Introduction

In cardiac muscle, the sarco(endo)plasmic reticulum Ca^2+^-ATPase (SERCA) is a crucial protein that catalyzes the transport of calcium ions (Ca^2+^) from the cytosol and into the sarcoplasmic reticulum (SR) using the energy from ATP hydrolysis. In doing so, SERCA initiates cardiac muscle relaxation while also recharging the SR with adequate [Ca^2+^] to be released for subsequent muscle contractions [1]. The body contains several SERCA isoforms with SERCA2a and SERCA2c being the dominant isoforms in cardiac muscle. This Ca^2+^ pump is responsible for 92% of Ca^2+^ removal from the cytosol in rodent heart and over 70% in human heart [1,2]. In the heart, the primary regulator of SERCA2 function is phospholamban (PLN). PLN exists in an inactive pentameric (storage) form and an active monomeric form that can bind to SERCA2 and reduce its affinity for Ca^2+^. This results in slower Ca^2+^ uptake into the SR, affecting the rate of relaxation and contraction [3,4].

Reactive oxygen and nitrogen species (RONS) can also regulate SERCA function as SERCA proteins are highly susceptible to oxidative and nitrosative damage that affects various tyrosine, cysteine, lysine, methionine, and histidine residues in the cytosolic and transmembrane domains [5]. These amino acids are susceptible to oxidation, nitration, and nitrosylation, which alter the SERCA structure and reduce Ca^2+^ binding and maximal ATPase function [5,6]. In mouse and human myocardium, increased cellular stress via heat stress [7], or direct application of RONS, significantly decreases SERCA2 pump function [8,9]. Considering SERCA2’s vital function in cardiac muscle performance, it is essential to preserve its function against oxidative stress through various mechanisms. Vulnerable cysteine residues on SERCA can be protected through S-glutathionylation, preventing oxidation of these cytosolic residues [10,11]. Additionally, the heat shock protein 70 (Hsp70) is up-regulated with increased RONS and has been shown to protect and even enhance SERCA activity during heat stress of HEK-293 cells and of fast-twitch skeletal muscles [5,7]. Transgenic overexpression of Hsp70 also preserved maximal SERCA function in SR vesicles isolated from *mdx* mice—a mouse model of Duchenne muscular dystrophy—following external application of increasing concentrations of peroxynitrite, demonstrating a potent protective effect [8].

The metabolic enzyme glycogen synthase kinase 3β (GSK3β) also plays a central role in the regulation of this system. GSK3β is known to be activated in conditions of high oxidative/nitrosative stress such as ischemia/reperfusion injury and oxygen glucose deprivation/reperfusion [12,13]. This enzyme is also known to negatively regulate SERCA2 content and function by preventing transcription of the *ATP2A2* gene [14,15] as well as Hsp70 content through inhibition of the transcription factor heat shock factor-1 (HSF-1) [16] and the antioxidant Nrf2 pathway [17]. We have shown that treating mice with sub-therapeutic lithium, an inhibitor of GSK3β, via lithium chloride (LiCl) supplementation [18], increased SERCA2 protein content while also reducing PLN content in the heart [19]. Interestingly, PLN has also been shown to provide a protective benefit to SERCA during thermal inactivation [20]; therefore, it is possible that LiCl supplementation could speed up the thermal inactivation of SERCA2 in the cardiac muscles by altering the SERCA2:PLN ratio. However, GSK3β inhibition with lithium treatment has also been shown to boost Hsp70 expression in neuronal [21,22], and cardiac tissue [23], suggesting there are two opposing effects that may influence SERCA2 thermal inactivation. The purpose of the present study was to explore whether GSK3β inhibition through low-dose LiCl supplementation would alter SERCA2’s susceptibility to thermal inactivation caused by heat stress in cardiac muscles obtained from male mice.

## Methods

### Animals and study design

Male 4-6 month old C57BL/6J mice (*n* = 6 per group) from Jackson Laboratory were supplemented with lithium in their drinking water for 6 weeks. Full animal procedures can be reviewed in Hamstra et al. [19]. To briefly summarize, mice were given sub-therapeutic lithium chloride (L4408; Sigma–Aldrich; St. Louis, MO, U.S.A.) via their drinking water at a dose of 10 mg/kg/day (serum [Li^+^] = 0.02 mM ± 0.004) for 6 weeks and control mice were given water without lithium supplementation [19]. Mice had free access to water and a standard chow diet (2014 Teklad global, 14% protein rodent maintenance diet, Harlan Teklad). Body weight, food intake, and water intake were measured twice a week. All procedures were approved by the Brock University Animal Care Committee (File #17-06-03) and in compliance with the Canadian Council on Animal Care. Mice were killed after 6 weeks of treatment by exsanguination under the administration of 5% isoflurane in oxygen. Left ventricles were isolated and frozen in liquid N_2_ immediately to be stored at –80°C. Tissue was homogenized in a 10:1 ratio with homogenizing buffer (250 mM sucrose, 5 mM HEPES, 0.2 mM phenylmethylsulfonyl fluoride, 0.2% [w/v] NaN3 in distilled H_2_O, pH 7.5). Each homogenate was aliquoted for the heat stress protocol (one aliquot for heating and one without heating).

### SERCA activity heat stress protocol

SERCA activity measured ATP hydrolysis indirectly through disappearance of NADH by an assumed ratio of 1:1 using an ionophore-supported spectrophotometric assay over a range of calcium concentration to be subtracted from values measured across a range of Ca^2+^ concentrations (*p*Ca 5–7). Kinetic assays were read at 340 nm for 30 min at 37°C using an M2 Molecular Devices MultiMode plate reader. SERCA-specific activity was determined after subtracting background ATPase activity from all calcium concentrations with a SERCA-specific inhibitor, cyclopiazonic acid (40 mM CPA dissolved in chloroform). Specifically, 1 μl of 40 mM cyclopiazonic acid (CPA) stock solution was added to a 300 μl SERCA activity reaction mixture (final CPA concentration 0.13 mM) containing homogenate, buffer, lactate dehydrogenase, pyruvate kinase, ionophore, and CaCl_2_ adjusted to *p*Ca 5.0. The ATPase activity measured in the presence of CPA was used to determine background ATPase activity, which was subtracted from SERCA activity measurements obtained at all other calcium concentrations within each sample. Full procedures can be reviewed here [19,24]. SERCA activity assays were repeated for each sample 30 and 60 min after incubating the homogenates at 40°C. After pathlength correction and using the extinction coefficient of NADH (6.22 mM), SERCA activity across the range of Ca^2+^ concentrations was normalized to grams of protein for each sample measured by the BCA assay. Maximal SERCA activity was then taken from the raw dataset.

### Western blotting

Prepared Western blot aliquots were made for both non-heated and 60 min heated samples with 5 μg of protein loaded for GSK3β, SERCA2, and PLN and 10 μg protein load for Hsp70 and pGSK3β. Western blots for SERCA2a, total and phosphorylated PLN (pPLN), Hsp70, phosphorylated and total GSK3α and GSK3β, and nuclear factor erythroid 2-related factor 2 (Nrf2) were performed using 7%–12% gradient BioRad TGX gels (4561086; BioRad, Hercules, CA, U.S.A.) and polyvinylidine difluoride (PVDF) membranes. After gel electrophoresis and protein transfer to the PVDF membranes, the membranes were blocked in 5% (w/v) milk in TBST for 1 h. Then, primary antibody was added (1:2000 dilution) and incubated overnight at 4°C. The primary antibodies for SERCA2a (MA3-919) and total PLN (MA3-922) were obtained from ThermoFisher Scientific (Waltham, MA, U.S.A.). The primary antibodies for phosphorylated GSK3β (Ser9; 9336), total GSK3β (9315), and Hsp70 (4872S) were obtained from Cell Signalling Technology (Beverly, MA, U.S.A.). After primary incubation, the membranes were washed three times with TBST at room temperature and then incubated with the corresponding anti-mouse (SERCA2a, t-PLN; 7076; Cell Signalling Technology) or anti-rabbit (Hsp70, p-/t-GSK3β; 7074; Cell Signalling Technology) HRP-conjugated secondary antibody (1:10000 dilution) for 1 h at room temperature. The membranes were washed again three times with TBST and then Millipore Immobilon Chemiluminescent Substrate (WBKLS0500; Sigma–Aldrich) was added to image the blots. Blots were imaged using a BioRad Chemi Doc Imager and protein expression was determined using BioRad ImageLab software and normalized to total protein using Ponceau S stain (59803; Cell Signalling Technology).

### SERCA2a immunoprecipitation

SERCA2a was isolated via immunoprecipitation to measure SERCA interactions with Hsp70 and glutathione. SERCA2a primary antibodies (5 μg) were coupled with 50 μg of SureBeads Protein G Magnetic beads (1614023; BioRad) in phosphate buffered saline tween (PBST) with a 15 min incubation at room temperature. The antibody–bead complex was then incubated with 100 μg of muscle homogenate in PBST for 2 h at room temperature. After, anything unbound to the antibody–bead complex was removed with three PBST washes. The immunoprecipitated (IP) SERCA2a was eluted with 60 μl of 1× non-reducing Laemmli buffer at 70°C for 10 min The eluent was used for Western blot analyses with 5 μl of SERCA2 eluent for glutathione (MA1-7620; ThermoFisher), Hsp70, and SERCA2 that were performed using 7%–12% gradient BioRad TGX gels and transferred onto nitrocellulose membranes. All blots were normalized to protein load using their corresponding ponceau stains. Hsp70 and glutathione levels were normalized to the amount of SERCA2a measured in the eluent after Western blotting.

### Statistical analysis

All experiments, with the exception of Nrf2 Western blot, were analyzed using a two-way repeated measures ANOVA to compare the main effects of LiCl supplementation, heat stress, and their potential interaction. Significant interactions were pursued with a Tukey’s *post-hoc* test. Nrf2 protein content was analyzed using an independent two-tailed *t*-test. All statistical tests were conducted using GraphPad Prism 8 Software and a *P* ≤0.05 defined statistical significance with trending significance defined as *P* <0.10. Outliers were identified using ROUT at 5% and were removed when identified. All data are presented as mean ± SEM.

## Results

**Figure 1:**
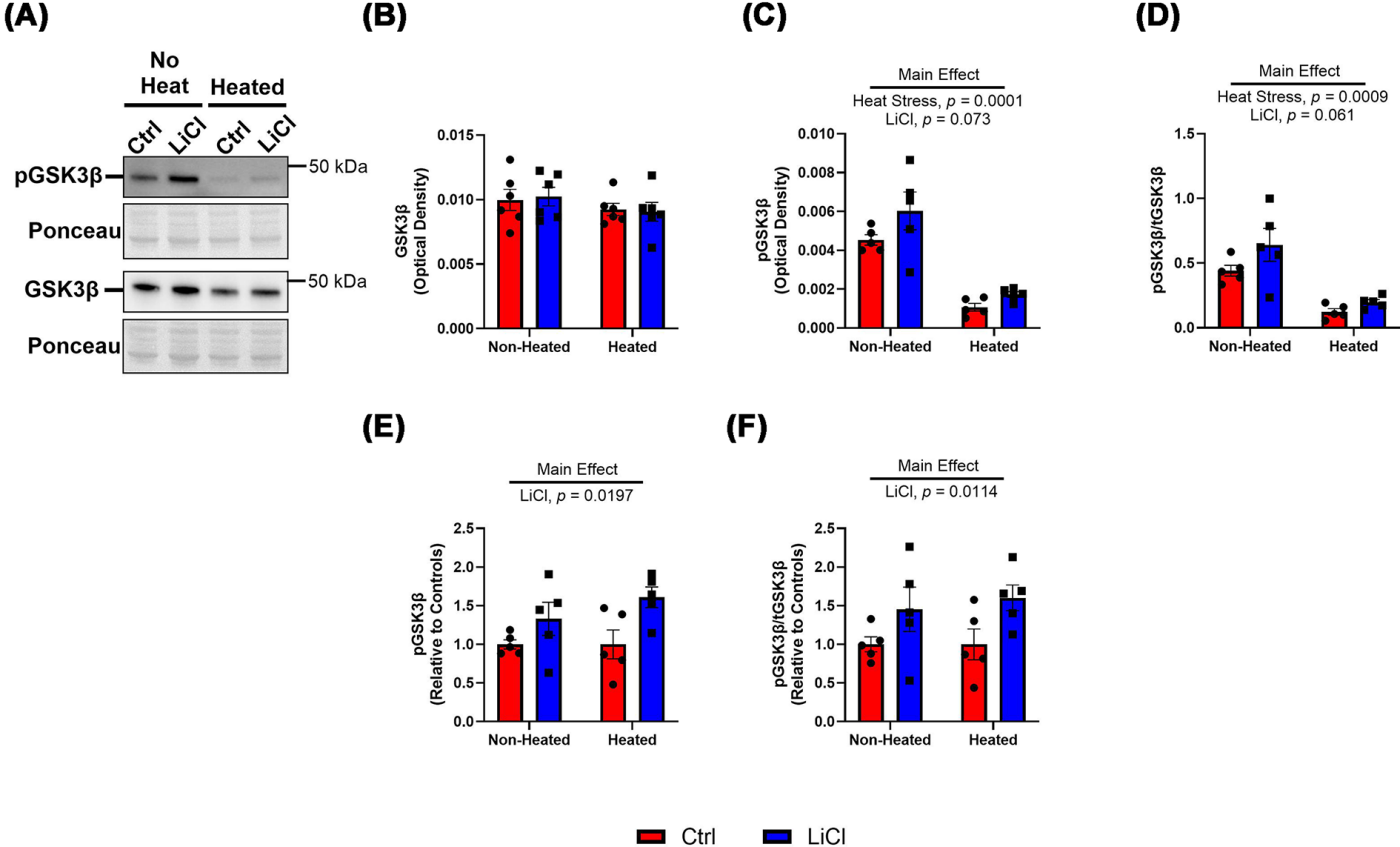
Low-dose lithium treatment preserves GSK3β phosphorylation during heat stress Representative Western blot images with corresponding ponceaus of non-heated and heated aliquots of total and Ser9 phosphorylated GSK3β. (**A**) Total GSK3β levels quantified by their optical density in non-heated and heated aliquots. (**B**) Ser9 phosphorylated GSK3β levels quantified by their optical density in non-heated and heated aliquots. (**C**) Ratio of Ser9 phosphorylated GSK3β to total GSK3β. (**D**) Ser9 phosphorylated GSK3β protein content normalized to non-heated and heated controls. (**E**) Exploratory analysis of the ratio of Ser9 phosphorylated to total GSK3β content normalized to non-heated and heated controls. (**F**) All results are mean ± SEM, *n* = 6 per group.

Given that GSK3β is the most dominant GSK3 isoform in the left ventricle of the heart [25], we subsequently aimed to confirm that the lithium treatment influenced GSK3β activation. Lithium can inhibit GSK3β by activating PI3K/Akt, which in turn phosphorylates serine (Ser) 9 on GSK3β resulting in inhibition [16]. Figure 1 demonstrates no changes due to heat or lithium treatment in total GSK3β protein content (Figure 1A,B). Heat stress did reduce Ser9 phosphorylation both absolute and relative to total GSK3β protein (Figure 1C,D). Conversely, lithium treatment increased Ser9 phosphorylation of GSK3, both absolute and relative to total GSK3β protein, though this did not reach statistical significance (Figure 1C,D). We attribute the lack of statistical significance with lithium treatment to the strong main effect of heat stress, accounting for 72.77% and 59.70% of the variation in the two-way ANOVA models for absolute and relative total pGSK3β. Therefore, we performed an exploratory analysis removing the effect of heat stress and isolating the effect of lithium treatment by normalizing the dataset to control in non-heated and heated conditions. This revealed a significant main effect of lithium on raising Ser9 phosphorylation (Figure 1E,F). Altogether, these results show that heat stress reduces GSK3β Ser9 phosphorylation, whereas low-dose LiCl supplementation increases it, though the effect of heat stress is most dominant.

**Figure 2:**
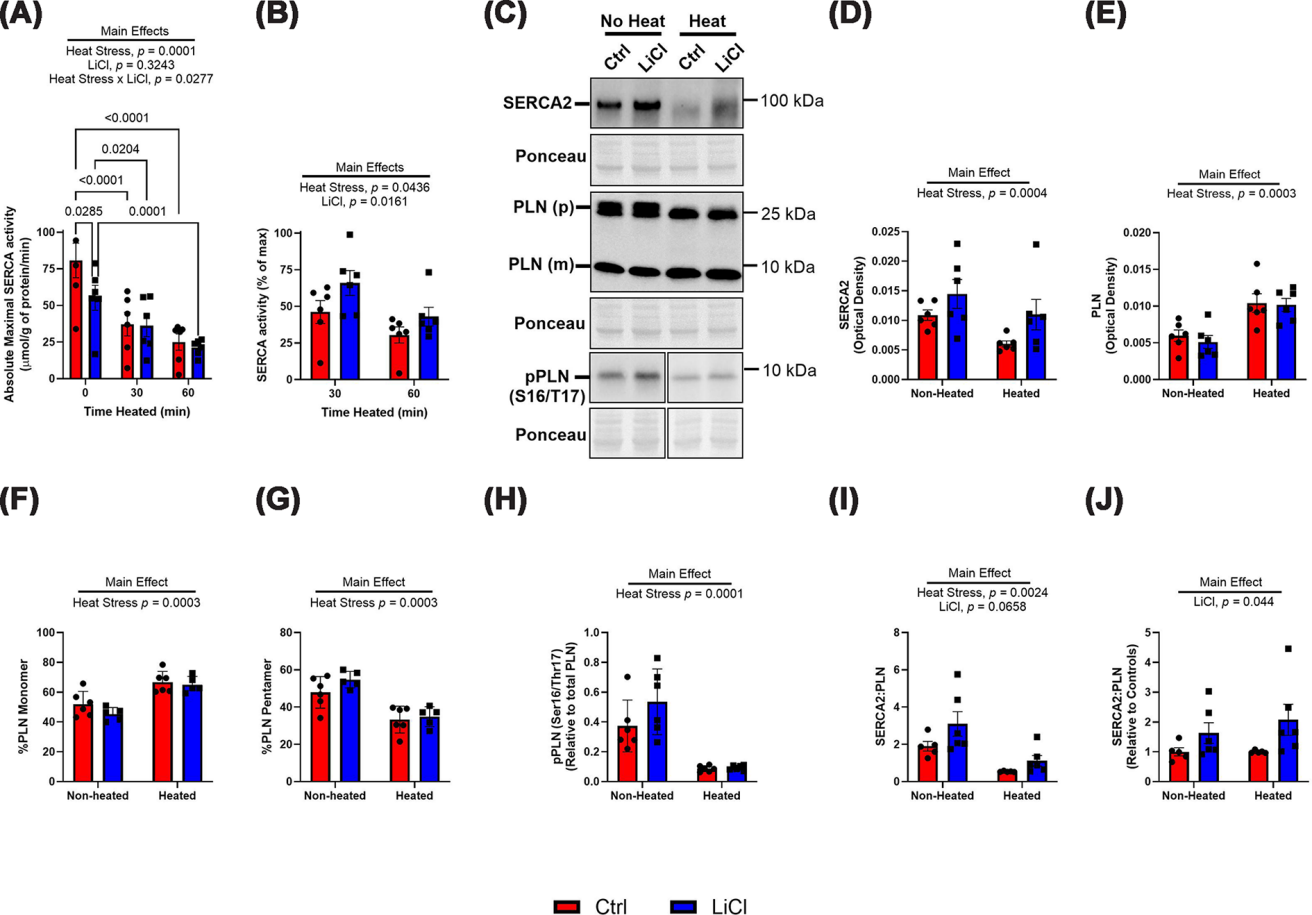
Maximal SERCA activity is preserved with low-dose lithium treatment during heat stress Absolute maximal SERCA-specific ATPase activity in the left ventricle in control and lithium-treated samples at baseline (0 min), and after 30 and 60 min of heating at 40°C. (**A**) SERCA-specific ATPase activity represented as a percent of *V*_max_ (max values at baseline) at 30 and 60 minute heating periods (**B**) Representative Western blot images of SERCA2 and PLN (monomer and pentamer), and Ser16/Thr17 pPLN with corresponding ponceau in non-heated and heated aliquots. (**C**) Quantified SERCA2 content in non-heated and heated aliquots. (**D**) Quantified monomeric PLN content in non-heated and heated aliquots. (**E**) Proportion of monomeric and pentameric PLN forms to total PLN content in non-heated and heated aliquots. (**F**,** G**) Ser16/Thr17 phosphorylated levels of PLN relative to total PLN in non-heated and heated aliquots. (**H**) Ratio of SERCA2 to monomeric PLN in non-heated and heated aliquots. (**I**) Exploratory analysis of the ratio of SERCA2 protein content to PLN protein content normalized to non-heated and heated controls. (**J**) Presented as mean ± SEM, *n* = 6 per group.

Next, SERCA activity, content, and PLN regulation were assessed with heat stress. Baseline maximal activity was lower in LiCl-treated LV homogenate compared with control (Figure 2A). When reporting the percent reduction after heat stress at both 30 and 60 min, significant main effects of both heat stress and LiCl are observed (Figure 2B). This suggests that extending heat stress caused further reductions in SERCA activity, with the percent reduction being less apparent with LiCl treatment. SERCA2 content was reduced with heat stress and although it appeared to be higher in both non-heated and heated conditions with LiCl, this was not statistically significant (non-heated Ctrl 0.011 ± 0.0009 vs. non-heated LiCl 0.014 ± 0.002 and heated Ctrl 0.006 ± 0.0005 vs. heated LiCl 0.011 ± 0.003, main effect of LiCl *P* = 0.115; Figure 2C,D). Heat stress also increased PLN monomer content (Figure 2C,E,F), which is the protein form that interacts with and inhibits SERCA2 [26] and is known to provide SERCA protection from thermal inactivation [20]. This was accompanied by a reduction in the percent of its pentameric form with heat stress (Figure 2C–G). However, there was no effect of LiCl as the amount of monomeric and pentameric PLN was comparable between control and LiCl groups, regardless of heat status (Figure 2C,E–G). Similarly, LiCl did not have an effect on inhibitory Ser16/Thr17 phosphorylation of monomeric PLN (Figure 2C–H); however, heat stress reduced pPLN levels (Figure 2C–H). Heat stress also reduced the ratio of SERCA2:PLN (monomer) with LiCl treatment appearing to increase this ratio; however, this effect did not reach statistical significance (Figure 2C–I). The lack of statistical significance with lithium treatment was again attributed to a relatively strong main effect of heat stress, accounting for 40.77% of the variance in the two-way ANOVA model. Therefore, we again performed an exploratory analysis by isolating the effect of lithium treatment by normalizing to controls in both non-heated and heated conditions that revealed a significant increase in SERCA2:PLN with LiCl treatment (non-heated Ctrl 1.00 ± 0.134 vs. non-heated LiCl 1.63 ± 0.347 and heated Ctrl 1.00 ± 0.02 vs. heated LiCl 2.08 ± 0.520, main effect of LiCl *P* <0.05; Figure 2J). Overall, the data indicate that although LiCl reduces baseline maximal SERCA activity, it can maintain SERCA activity under heat stress by preserving higher percent of baseline maximal SERCA activity compared with controls. Thus, the increase in SERCA2 relative to PLN found with LiCl supplementation does not accelerate the thermal inactivation of SERCA2 in the heart.

**Figure 3:**
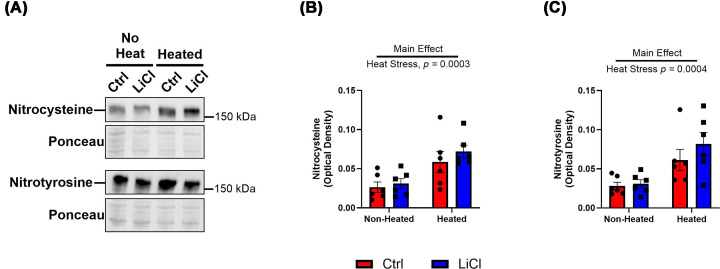
Heat stress increases total RONS residues Representative Western blot images with corresponding ponceaus of non-heated and heated aliquots of total nitrocysteine and nitrotyrosine residues. (**A**) Total nitrocysteine residues quantified by their optical density in non-heated and heated aliquots. (**B**) Total nitrotyrosine residues quantified by optical density in non-heated and heated aliquots. (**C**) All results are presented as mean ± SEM, *n* = 6 per group.

Previous studies looking at *ex vivo* heat stress of HEK-293 cells and skeletal and cardiac muscle homogenate have shown increases in residues modified by RONS [2,6,10]. Here, we wanted to confirm that *ex vivo* heat stress caused oxidative and nitrosative stress in the left ventricle cardiac muscle homogenates, as indicated by non-specific protein residue modifications. Figure 3 demonstrates this with a significant increase in cysteine nitration (nitrocysteine, Figure 3A,B) and tyrosine nitration (nitrotyrosine, Figure 3A–C). Lithium treatment did not have an effect on the levels of nitrocysteine or nitrotyrosine.

**Figure 4:**
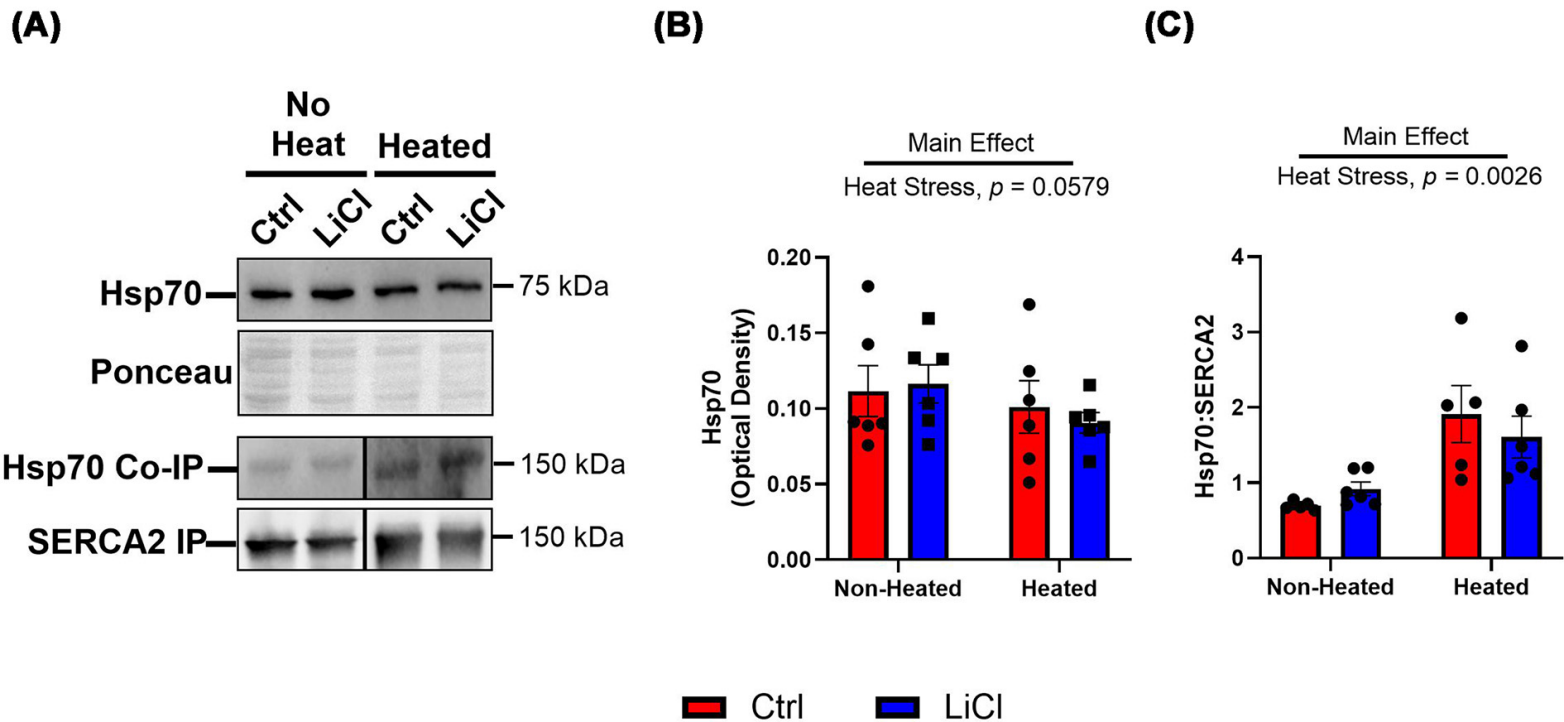
Heat stress increases Hsp70 binding to SERCA2 but low-dose lithium does not alter Hsp70 content or interaction with SERCA2 Representative Western blot images of total Hsp70 content, IP SERCA2, and co-immunoprecipitated (Co-IP) Hsp70 content in non-heated and heated aliquots with corresponding ponceau. (**A**) Total Hsp70 levels quantified by its optical density in non-heated and heated aliquots. (**B**) Levels of Hsp70 bound to isolated SERCA2 protein in non-heated and heated aliquots. (**C**) Presented as mean ± SEM, *n* = 6 per group.

We then quantified Hsp70 contents in the LV to investigate whether LiCl-induced up-regulation of this chaperone protein contributes to the observed preservation of SERCA function under heat stress conditions. A decreasing trend in Hsp70 content was seen with heat stress (non-heated Ctrl 0.112 ± 0.017 vs. heated Ctrl 0.101 ± 0.017 and non-heated LiCl 0.116 ± 0.013 vs. heated LiCl 0.091 ± 0.007, main effect of heat stress *P* = 0.0579; Figure 4A,B). SERCA2 was also isolated by immunoprecipitation, and the amount of Hsp70 bound was measured thereafter. An increase in Hsp70 bound to SERCA2 was observed with heat stress. However, LiCl did not appear to affect total Hsp70 content or the amount of Hsp70 bound to SERCA2 (Figure 4A–C).

**Figure 5:**
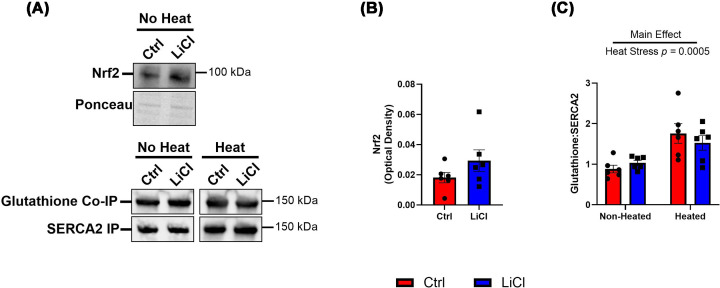
Low-dose lithium does not appear to affect Nrf2 content or glutathione activity Representative Western blot images of Nrf2 in non-heated aliquots and IP SERCA2 with Co-IP glutathione in non-heated and heated aliquots. (**A**) Total Nrf2 content was quantified by optical density in non-heated samples. (**B**) Quantified glutathione bound to isolated SERCA2 in non-heated and heated aliquots. (**C**) Presented as mean ± SEM, *n* = 6 per group.

To further explore the potential underlying mechanisms behind the preservation of SERCA activity with thermal inactivation, we next explored the effects of LiCl on Nrf2 levels and glutathione (GSH) protection. GSK3 is a negative regulator of Nrf2 [27], a transcription factor for antioxidant enzymes such as glutathione s-transferase, glutathione reductase, and glutathione peroxidase, which regulate GSH metabolism [28]. GSH is a tripeptide [29] capable of increasing SERCA activity and preventing oxidative damage to its cytosolic domain by binding to the vulnerable Cys674 residue [10]. We wanted to determine whether low-dose LiCl was capable of affecting this antioxidant pathway to provide protection to SERCA2 during heat stress. However, Figure 5 demonstrates no effect of LiCl treatment on Nrf2 content in non-heated muscle homogenate (Figure 5A,B). When observing the amount of glutathione bound to isolated SERCA2, heat stress appeared to increase glutathione binding; however, LiCl did not have an effect on this interaction (Figure 5A–C).

## Discussion

The purpose of the present study was to explore the possible influence of low-dose lithium supplementation (with LiCl) on SERCA2 inactivation in male cardiac muscle exposed to heat stress. In response, we found several processes that act to preserve SERCA2 function during heat stress, including increased monomeric PLN, reduced inhibitory phosphorylation of PLN, and increased SERCA2 binding of Hsp70 and glutathione. However, neither of these effects was influenced by LiCl supplementation, despite LiCl treatment showing preservation of maximal SERCA activity in response to heat stress.

Given our previous findings of the inhibitory effects of lithium on GSK3β activity and its downstream positive influence on SERCA function, we assessed GSK3β content and its inhibition through Ser9 phosphorylation. Here, we found a significant reduction in phosphorylation levels but no changes in total GSK3β levels with heat stress (Figure 1). This suggests that heat stress could be particularly affecting the N-terminal inhibitory domain where Ser9 is located in GSK3β. Previous work has shown that *ex vivo* heat stress of skeletal muscle increases the activity of μ-calpain (CAPN1) due to increases in intracellular Ca^2+^ concentrations [30]. CAPN1 is capable of cleaving the Ser9 site of GSK3β [31]; therefore, we speculate that increased cytotoxic stress from heating the samples at 40°C for 60 min could be increasing CAPN1 activity, causing a reduction in Ser9 phosphorylation without changes in total GSK3β content; however, this remains to be further investigated.

Regardless of the exact mechanism, the effect of heat stress on GSK3β Ser9 phosphorylation was significant, such that the anticipated effect of LiCl on increasing Ser9 phosphorylation of GSK3β was statistically obscured and could only be observed when removing the effect of heat stress (Figure 1E,F) although we acknowledge that this is only a correlative indicator. Given that acute lithium treatment has also been shown to reduce calpain activity in a neonatal rat model of hypoxia/ischemia of the brain [32], potentially through improved intracellular Ca^2+^ regulation [33], we speculate that lithium’s preservation of Ser9 phosphorylation, especially in the absence of a phosphatase inhibitor, could also be due to a potential reduction in calpain activity during the *ex vivo* heating process and should be examined further in muscle tissue.

With respect to SERCA2, prolonged heat stress can reduce maximal SERCA activity in cardiac and skeletal muscle homogenate [7,20]. In the present study, low-dose LiCl treatment preserved maximal SERCA activity during heat stress at 40°C (Figure 2). Although the LiCl group showed lower baseline maximum SERCA activity than controls, it experienced smaller percentage reductions at both 30 and 60 min of heating (Figure 2B). We acknowledge that this apparent preservation could result from the lower starting ATPase activity in the LiCl group, potentially inflating the percentage change. However, if heat stress affected both groups equally, the absolute declines in activity would be similar between control and LiCl-treated samples. However, this was not observed. On average, control samples declined by 43.5 ± 7.7 μmol/g protein/min from 0–30 min and 55.8 ± 8.2 μmol/g protein/min from 0–60 min, whereas LiCl-treated samples declined by only 19.1 ± 5.7 μmol/g protein/min from 0–30 min (*P* = 0.030 vs. control, Student’s *t*-test) and 33.9 ± 6.7 μmol/g protein/min from 0–60 min (*P* = 0.066 vs. control, Student’s *t*-test). Therefore, even in absolute terms, LiCl-treated samples showed a smaller decrease in maximal SERCA activity under heat stress. When expressed relative to baseline, this smaller absolute decline—paired with the lower initial activity—leads to the higher percentage preservation observed in the LiCl group (Figure 2B). Importantly, because the absolute decline itself is smaller, we view the conclusion that LiCl treatment preserves maximum SERCA activity during heat stress to be valid.

The mechanism behind the baseline reduction in maximal SERCA activity with LiCl treatment is currently unknown; however, it is not due to any decreases in SERCA2 content, which is a key factor in determining maximal SERCA activity [34]. It is possible that LiCl treatment could have caused additional post-translational modifications of SERCA, acting on GSK3-dependent or independent pathways, which might negatively impact maximal SERCA activity; however, this needs further investigation. Nonetheless, we do not believe this to be a pathologically significant effect. In a previous study using the same dose of LiCl over 12 weeks [35], we observed no impairments in systolic or diastolic function, despite noting a similar trend toward reduced maximal SERCA activity. Instead, LiCl-treated mice exhibited increased stroke volume and an enlarged LV internal diameter without changes in wall thickness, consistent with physiological hypertrophy.

The preservation of SERCA function during heat stress with LiCl supplementation took place with a potential increase in the SERCA2:PLN ratio in the heart (significant main effect when removing the effect of heat stress; Figure 2I). This increase in SERCA2:PLN content is consistent with our previous findings in male C57BL/6J mice, where we found that a 10 mg/kg/day dose of LiCl for 6 weeks increased SERCA2 Ca^2+^ sensitivity and SERCA2:PLN content [19]. Previous work has shown that HEK-293 cells co-transfected with SERCA2 and PLN exhibit preserved maximal SERCA activity compared with cells expressing SERCA2 alone during heat stress [20]. The same work also shows that this protection is lost when comparing maximal SERCA activity in the left ventricle of *Pln-*null mice to that of their control counterparts [20]. Thus, PLN is known to protect SERCA2 from thermal inactivation in the heart. Here, we found that heat stress significantly increased the amount of monomeric PLN (Figure 2E) and reduced inhibitory phosphorylation of the monomer (Figure 2H)—the form of PLN that is able to bind to SERCA2. While heating mouse left ventricle and atria homogenate has been known to cause the dissociation of PLN pentamers into the monomeric form [36], we also view this to be an adaptive mechanism in response to heat stress aimed at preserving SERCA activity. This effect is further bolstered by the reduction in inhibitory phosphorylation of PLN with heat stress allowing for binding of the monomeric form to PLN, an effect that has also been observed in other models of cardiac stress including ischemia/reperfusion injury induced by coronary artery occlusion in rats [37] and in the failing myocardium of rats [38] and humans [39]. One could suspect that by lowering the amount of PLN relative to SERCA2 (or raising SERCA2:PLN levels, Figure 2I), LiCl may accelerate the thermal inactivation of SERCA2 in the heart. However, this was not observed, and in fact the opposite was found.

*Ex vivo* heat stress led to significant increases in total protein nitrocysteine (Figure 3A,B) and nitrotyrosine residues (Figure 3A–C). However, our dose of LiCl did not have an effect on these residues. To try to explain the preservation of SERCA activity with LiCl treatment, we next looked at Hsp70 content. With LiCl treatment, we did not see any change in total Hsp70 content or binding to SERCA2; however, we did see increased binding to SERCA2 content with heat stress (Figure 4). Though there were no changes with LiCl supplementation, this represents another adaptive mechanism aimed at preserving maximal SERCA activity during heat stress, as Hsp70 has been shown to stabilize the nucleotide-binding domain of SERCA2 and preserve its activity during heat stress and high cytotoxic stress [5,7,8].

We next examined the Nrf2 pathway, which transcribes several antioxidant enzymes such as glutathione s-transferase [40] that catalyzes the interaction between glutathione (GSH) and electrophilic centers of proteins to protect them from oxidative damage [41]. GSK3 is known to negatively regulate Nrf2 signaling and lithium-mediated GSK3 inhibition has been shown to increase Nrf2 in drosophila, as well as in rodent and mammalian cell lines [27,42,43]. GSH has been proven to bind to and protect the SERCA proteins from oxidative damage. This happens through reversible binding of GSH to the Cys674 residue on the cytosolic portion of SERCA2, enhancing maximal ATP-dependent Ca^2+^ uptake [44]. However, in the present study, no changes were observed for Nrf2 or GSH with LiCl treatment (Figure 5A,B). When looking at GSH bound to SERCA2, there was a significant increase with heat stress, which is to be expected with increased nitrocysteine and nitrotyrosine residues that are also seen here (Figure 3); however, there was no effect of LiCl (Figure 5A–D). Thus, while low-dose LiCl supplementation preserves maximal SERCA activity during heat stress, the exact mechanisms remain unknown.

One possible mechanism involves the direct phosphorylation of SERCA2 by GSK3. Previous work by Gonnot et al. demonstrated that SERCA2 can be phosphorylated at Ser663, a modification that reduces Ca^2+^ uptake into the SR and becomes elevated during hypoxia/reoxygenation injury in HEK 293 cells—a model of increased oxidative and nitrosative stress [45]. They further identified GSK3β as a likely kinase responsible for this phosphorylation, as it can bind to SERCA2 and localize to the SR membrane. Supporting this interaction, their FRET-based assays show that GSK3β and SERCA2 exhibit increased colocalization under conditions of oxidative stress [45], suggesting that stress-induced proximity may facilitate GSK3β-dependent inhibitory phosphorylation of SERCA2. Thus, we speculate that the preservation of maximal activity found here with LiCl treatment could be due to less GSK3β-mediated phosphorylation of SERCA2 and should be tested in the future when phosphor-specific antibodies become readily available. Furthermore, GSK3β is a node kinase in numerous metabolic and survival signaling pathways; therefore, its inhibition may alter the cellular response to heat stress or indirectly affect the stability of SERCA by influencing a broader signaling network resulting in a protective mechanism that is yet to be identified.

Some considerations for the present work in terms of limitations were that only male mice were used. Young adult females tend to have a greater resilience to oxidative stress compared with males [46]; therefore, it would be interesting to determine whether low-dose LiCl treatment would provide the same effect in the myocardium of female mice. We also acknowledge that there are many post-translational modifications that can affect SERCA2 protein [1] and that LiCl could have exerted a protective effect on. However, this would have required comprehensive proteomic analysis; therefore, we chose nitrotyrosine and nitrocysteine residues that are easily probed for and known to be affected by this heat stress protocol [5,20]. We also acknowledge that phosphatase inhibitors were not included in the homogenization buffer, as these compounds interfere with SERCA activity measurements performed using the same homogenates [47]. While the absence of phosphatase inhibitors could theoretically influence phosphorylation measurements, all samples were processed identically; therefore, any potential reduction in phosphorylation would be expected to affect control and LiCl groups equally and would not bias comparisons between groups.

Further, we understand that this model of cellular stress is not a physiological model of stress such as ischemia/reperfusion (I/R) injury. The use of the *ex vivo* heat stress model was used as a proof-of-concept model to determine whether this sub-therapeutic dose of lithium could provide any protection to SERCA maximal capacity for ATPase activity when under stress via thermal inactivation. Previous study has already shown that both acute and chronic lithium treatment at therapeutic concentrations improves recovery of ventricular function and reduces infarct size post-I/R injury [48]; however, this has not been investigated with a sub-therapeutic dose. A logical next step would be to determine cardiac SERCA function before and after myocardial I/R injury or another model of murine heart failure with sub-therapeutic LiCl feeding before or after insult to determine a physiological protective effect.

In conclusion, our study examined the effects of LiCl on thermal inactivation of SERCA in cardiac muscles from male mice. Both untreated and LiCl-treated hearts experienced thermal inactivation; however, the reduction in maximal SERCA activity was greater in the control group. Though the exact mechanisms remain unclear, the inhibitory effect of LiCl on GSK3β may be at play, potentially by limiting Ser663 phosphorylation to SERCA; however, this requires further investigation. Additionally, our findings also show that in response to heat stress several adaptive mechanisms may be active that aim to limit SERCA inactivation, including an increase in PLN monomers and increased Hsp70 and GSH binding to SERCA2. Though neither of these effects was influenced by LiCl, studies using higher doses may reveal an effect.

## Data Availability

All data pertaining to the manuscript can be made available upon reasonable request to the corresponding author.

## Abbreviations

BCA: Bicinchoninic Acid
CPA: cyclopiazonic acid
GSK3β: glycogen synthase kinase 3β
HSF-1: heat shock factor-1
Hsp70: heat shock protein 70
IP: immunoprecipitated
LV: Left ventricle
Nrf2: nuclear factor erythroid 2-related factor 2
PBST: phosphate buffered saline tween
PLN: phospholamban
pPLN: phosphorylated PLN
PVDF: polyvinylidine difluoride
RONS: reactive oxygen and nitrogen species
SERCA2: sarco(endo)plasmic reticulum calcium (Ca2+)-ATPase 2
SR: sarcoplasmic reticulum
TBST: Tris-Buffered Saline with Tween 20
